# 1473. Procedural risk factors for deep and organ/space surgical site infection post-coronary artery bypass graft surgery

**DOI:** 10.1093/ofid/ofad500.1309

**Published:** 2023-11-27

**Authors:** Abarna Pearl, Patrick S Gordon, Baevin Feeser, Dana Pepe, Preeti Mehrotra, Sharon B Wright

**Affiliations:** Beth Israel Deaconess Medical Center, Boston, Massachusetts; Beth Israel Deaconess Medical Center, Boston, Massachusetts; Beth Israel Deaconess Medical Center, Boston, Massachusetts; Beth Israel Deaconess Medical Center/Harvard Medical School, Boston, Massachusetts; Beth Israel Deaconess Medical Center/Harvard Medical School, Boston, Massachusetts; Beth Israel Lahey Health, Cambridge, Massachusetts

## Abstract

**Background:**

Advances in coronary artery bypass graft (CABG) surgery have improved graft patency and survival. Our prior work suggested a reduction in deep and organ/space surgical site infection (SSI) with single versus bilateral internal mammary arteries and a skeletonized approach. Even for the same number of arterial grafts used, specific artery type may impact SSI incidence. We aim to assess the effect of potentially modifiable risk factors on post-CABG deep and organ/space SSI, including number and type of vessel grafts, and closure method.

**Methods:**

This was a single-center retrospective cohort study of post-CABG surgery patients from 01/01/19-12/31/22. SSIs were identified during routine surveillance using National Healthcare Safety Network criteria. Data on demographics, comorbidities, surgical technique and surgeon were obtained from hospital and cardiac surgery databases. A sub-analysis evaluated the risk of SSI in patients with two arterial grafts by type of artery used, specifically bilateral internal mammary (BIMA) graft versus single internal mammary artery plus radial graft (SIMA-R). We performed univariate analyses to determine association of risk factors with SSI.

**Results:**

Of the 2050 included patients, 23 developed an SSI . In univariate analyses, diabetes mellitus (OR 5.6, p < 0.001) and negative pressure wound therapy (NPWT) versus surgical adhesive (OR 19.82, p< 0.001) were associated with SSI. There was no significant association between SSI and the total number of arteries or combination of vessel grafts used. In the sub-analysis, 281 patients underwent SIMA-R and 154 underwent BIMA. None of the SIMA-R, versus 4 of the BIMA developed SSI (p=0.02). There were other differences between the groups, including a greater proportion with a skeletonized (versus pedicled) arterial harvest (OR 2.19, p=0.003) and longer case duration (238 vs 217 minutes, p=0.003) in the BIMA group, which could confound this relationship.

**Conclusion:**

In the overall cohort, NPWT was associated with SSI on univariate analysis, though notably this closure method is often used in patients with higher pre-operative risk. Amongst those patients who received two arterial grafts, there was a reduced SSI risk with SIMA-R approach. Future studies should explore the impacts of graft choice and wound care.

**Disclosures:**

**All Authors**: No reported disclosures

